# Assays to Monitor Autophagy in *Saccharomyces cerevisiae*

**DOI:** 10.3390/cells6030023

**Published:** 2017-07-13

**Authors:** Raffaela Torggler, Daniel Papinski, Claudine Kraft

**Affiliations:** Max F. Perutz Laboratories, University of Vienna, Vienna Biocenter (VBC), Dr.-Bohr-Gasse 9, 1030 Vienna, Austria

**Keywords:** yeast, bulk autophagy, Cvt pathway, selective autophagy, PAS, autophagosome, autophagic body, iPass, M-Track

## Abstract

Autophagy is an intracellular process responsible for the degradation and recycling of cytoplasmic components. It selectively removes harmful cellular material and enables the cell to survive starvation by mobilizing nutrients via the bulk degradation of cytoplasmic components. While research over the last decades has led to the discovery of the key factors involved in autophagy, the pathway is not yet completely understood. The first studies of autophagy on a molecular level were conducted in the yeast *Saccharomyces cerevisiae*. Building up on these studies, many homologs have been found in higher eukaryotes. Yeast remains a highly relevant model organism for studying autophagy, with a wide range of established methods to elucidate the molecular details of the autophagy pathway. In this review, we provide an overview of methods to study both selective and bulk autophagy, including intermediate steps in the yeast *Saccharomyces cerevisiae*. We compare different assays, discuss their advantages and limitations and list potential applications.

## 1. Introduction

**Figure 1:**
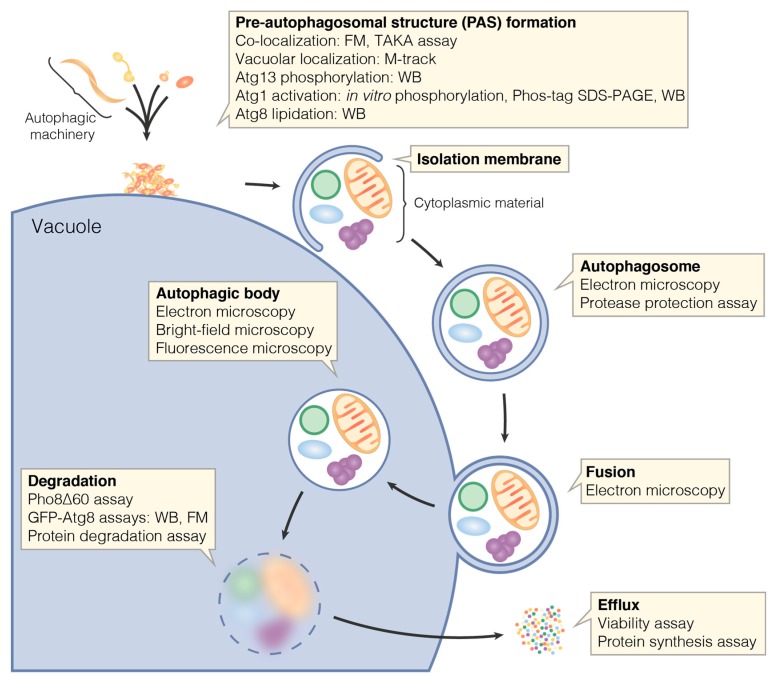
Methods for studying individual steps of bulk autophagy. Bulk autophagy and selective types of autophagy largely rely on the same core machinery. Many of the assays depicted up to vacuolar degradation are also useful for studying selective autophagy—see text for details. FM: fluorescence microscopy; WB: Western blotting.

Macroautophagy, hereafter referred to as autophagy, is an intracellular degradation and recycling pathway highly conserved among eukaryotes. During autophagy, cellular material is engulfed by a so-called isolation membrane resulting in the formation of a double membrane vesicle, the autophagosome. The outer autophagosomal membrane fuses with a lytic compartment, that is, a lysosome in mammals or the vacuole in yeast and plants. This releases the inner vesicle into the lytic compartment’s lumen. The inner vesicle—now termed autophagic body—and its contents are degraded by the resident hydrolases, and the catabolites are exported to the cytoplasm and recycled (Figure 1) [1]. 

Autophagy ensures cellular homeostasis and function by selectively degrading superfluous, damaged or harmful cytoplasmic components, including whole organelles and large protein aggregates. Not surprisingly, malfunctions in the autophagic pathway have been linked to human diseases such as neurodegenerative disorders or cancer [2]. During these selective types of autophagy, cargo receptors recognize the material to be degraded—the cargo—and link it to the autophagic machinery [3]. This leads to the selective and exclusive engulfment of the cargo by an isolation membrane [4,5]. Autophagy can not only target specific cargoes but can also degrade random cytoplasmic material to mobilize nutrients in stress conditions such as starvation. Thus, autophagy is not only required for cellular homeostasis but also for cell survival during nutrient deprivation [6].

The term autophagy was coined already in the 1960s by Christian De Duve [7,8]. However, the first investigation of autophagy on a molecular level was performed in the 1990s in the yeast *Saccharomyces cerevisiae*, leading to the discovery of 15 autophagy related genes (*ATG*) [9]. Today, more than 40 Atg proteins involved in different steps of the autophagic pathways have been described. A set of Atg proteins, commonly referred to as core autophagic machinery, is required for both selective and bulk autophagy, while other Atg proteins are specific for either type [10]. For many yeast autophagy proteins, homologs have been identified in higher eukaryotes and many mechanisms during the autophagic pathway have been found to be conserved [11]. 

## 2. Yeast as a Model Organism to Study Autophagy

Genetic screens in yeast resulted in the discovery of the first autophagy related proteins [9,12,13]. The identification of Atg proteins has allowed researchers to study the mechanisms of autophagy in yeast in molecular detail, making yeast the model organism in which the molecular details of the autophagy pathway are understood best. This work was pioneered by Yoshinori Ohsumi, who was awarded the 2016 Nobel Prize in Physiology or Medicine for discovering molecular mechanisms of autophagy.

In yeast, bulk autophagy is inhibited when external nutrients are available. Several intracellular pathways sense nutrient availability. The central nutrient sensing pathway signals through target of rapamycin complex 1 (TORC1) [14]. Inhibition of TORC1 by nutrient deprivation or rapamycin treatment induces bulk autophagy [15]. In addition to the TORC1 pathway, the PKA and Snf1/AMPK pathways have been found to play a role in the induction of bulk autophagy [16,17,18].

An early step in yeast autophagy involves the assembly of Atg proteins at the vacuolar membrane, where they form the pre-autophagosomal structure (PAS) [19]. This core autophagic machinery can be divided into different subgroups: The Atg1 kinase complex, the transmembrane protein Atg9, the autophagy specific phosphatidylinositol 3-kinase complex, the Atg12 conjugation system, the Atg8 conjugation system and the Atg2/Atg18 complex [10]. The interplay between the different subgroups is important during the autophagic pathway. For example, the phosphorylation of Atg9 by Atg1 is required for the elongation of the isolation membrane [20].

The Cytoplasm-to-vacuole targeting (Cvt) pathway is a special, biosynthetic form of selective autophagy in yeast. The Cvt pathway selectively transports the cytoplasmic proteins aminopeptidase 1 (Ape1), α-mannosidase 1 (Ams1) and aspartyl aminopeptidase 4 (Ape4) to the vacuole [13,21,22,23]. It uses the core autophagic machinery and is constitutively active also under nutrient-rich conditions [4]. Because of these features, the vacuolar delivery of the main Cvt cargo, Ape1, is used as a model pathway for selective autophagy. Ape1 is synthesized in the cytoplasm as an inactive precursor (preApe1). When transported to the vacuole it is not degraded but proteolytically processed to its mature and active form (mApe1) to function as a vacuolar hydrolase. 

As autophagy is a highly-conserved pathway, discoveries in yeast set the basis for studies in mammalian cells and for applied medical research. Although mammalian homologs for many yeast Atg proteins have been identified, yeast still remains an ideal model organism to study autophagy in vivo. A vast array of biochemical, cell biological and genetic approaches for research in yeast are available. Large amounts of yeast can be grown to purify native protein complexes in high quantities for biochemical or structural analyses [24]. Genetic screens have been performed extensively in yeast, including whole genome screens for synthetic lethality [25]. Such screens and yeast research in general benefit from well-established methods for the fast and simple manipulation of the yeast genome. The yeast genome is fully sequenced, and genome databases provide information about gene sequence and function. Genes can be easily modified or deleted by homologous recombination [26]. Although gene editing approaches such as CRISPR/Cas9 allow a relatively simple manipulation of mammalian genomes [27,28], yeast offers several advantages over mammalian systems. As yeast can be cultured in a haploid form, only one allele needs to be targeted for gene modification. In addition to genome manipulation, plasmid based expression of proteins is well established in yeast. The regulation of the gene expression level is robust and simple. Genes can be overexpressed using a high copy plasmid or a strong, constitutive or inducible promoter [29,30]. In addition, genes can also be expressed at endogenous levels using a centromere (CEN) plasmid under the control of its endogenous promoter. CEN plasmids, containing a yeast centromere region coupled to an autonomously replicating sequence, are replicated as an independent chromosome [31,32].

Yeast offers additional advantages specific for studying autophagy. Bulk autophagy is completely inhibited in nutrient-rich conditions, but can be induced rapidly either by shifting cells to starvation medium or by directly inhibiting TORC1 by rapamycin treatment [15,33]. The constitutively active Cvt pathway represents an ideal model to study selective autophagy in nutrient-rich, logarithmic growth conditions when bulk autophagy is inhibited. Thus, selective and bulk autophagy can be investigated separately by controlling the nutrient availability. 

As yeast is a widely-used model organism for autophagy, many methods to monitor autophagy are well established. This review provides an overview about these methods and discusses their advantages and limitations.

## 3. End-Point Measurements

Functional autophagy results in the delivery of cellular material to the vacuole and the exposure of this material to vacuolar enzymes. In this review, we refer to methods monitoring delivery to or degradation in the vacuole as end-point measurements. Such end-point measurements offer a sensitive readout for the overall function of autophagic pathways, but do not provide information on the individual function of single steps in these pathways. Methods to investigate individual steps of autophagic pathways are discussed in a separate section and are shown in Figure 1. 

As the degradation of autophagic material depends on the activity of vacuolar proteases, end-point assays measuring degradation are limited to yeast strains in which these proteases are functional. End-point assays monitoring the delivery of autophagic material to the vacuole rather than its degradation are generally independent of the activity of vacuolar proteases.

We define the end-point of autophagy as the delivery of material to and its degradation in the vacuole. One can argue, however, that the final step of autophagy is the efflux of catabolites to the cytoplasm after the degradation of autophagic cargo in the vacuole. While we rather regard catabolite efflux as a general vacuolar function, we also present methods to measure catabolite efflux from the vacuole in this review.

### 3.1. End-Point Measurements to Monitor Bulk Autophagy

Bulk autophagy is induced when nutrient supply becomes limited. Thus, bulk autophagy is gradually induced when cells reach stationary phase. Experimentally, abrupt induction can be achieved by shifting cells to nitrogen starvation medium or by inhibiting TORC1 by rapamycin treatment. Methods to monitor bulk autophagy often rely on a signal that accumulates over time after bulk autophagy induction. Such a signal will also accumulate if cells reach stationary phase before the actual experiment is performed. To ensure that a pre-accumulated signal does not influence the obtained results, the cells need to be cultivated overnight in logarithmic phase before experimentally inducing bulk autophagy. 

#### 3.1.1. The Pho8∆60 Assay (ALP Assay)

**Figure 2:**
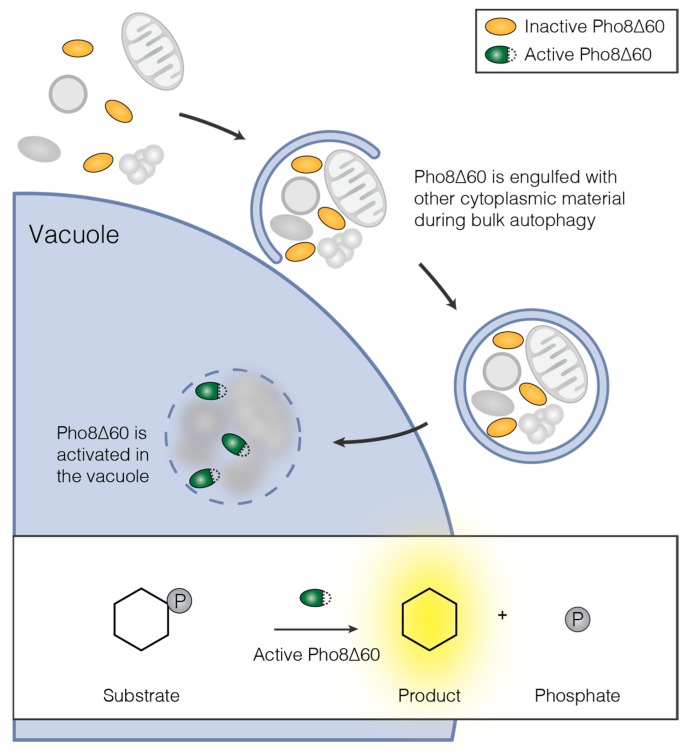
Principle of the Pho8∆60 assay for measuring bulk autophagic flux. The inactive precursor of Pho8∆60 is randomly sequestered in bulk autophagosomes together with cytoplasmic material. Upon delivery to the vacuole, Pho8∆60 is proteolytically activated. Insert: The activity of Pho8∆60 is measured in cell lysate by supplying a phosphatase substrate. Depending on the substrate, dephosphorylation by Pho8∆60 yields products, which can be detected either spectrophotometrically or spectrofluorometrically—see text for details.

The Pho8∆60 assay is a quantitative enzymatic assay to monitor bulk autophagy (Figure 2) [34]. The alkaline phosphatase (ALP) Pho8 is synthesized as an inactive precursor, which translocated to the endoplasmic reticulum (ER). From the ER, it is transported via the secretory pathway to the vacuole, where it is activated by proteolytic processing. The deletion of amino acids 1–60 of Pho8 (Pho8∆60) prevents its translocation into the ER. Thus, Pho8∆60 remains in the cytosol in its inactive form. Following the induction of bulk autophagy, Pho8∆60 is randomly engulfed in bulk autophagosomes, resulting in its transport to and activation in the vacuole. Therefore, Pho8∆60 phosphatase activity is a readout for bulk autophagy function. It can be measured by assessing the ability of Pho8∆60 cell lysate to dephosphorylate substrates such as para-nitrophenyl phosphate or α-naphthyl phosphate (Figure 2) [15,34]. 

The conversion of colorless para-nitrophenyl phosphate to yellow para-nitrophenol can be measured spectrophotometrically as an increase in the absorption at 400 nm [35]. The fluorogenic substrate α-naphthyl phosphate is dephosphorylated to α-naphthol by Pho8∆60 [15]. When excited at 345 nm, α-naphthol emits light at 472 nm. Both para-nitrophenol absorbance and α-naphthol fluorescence can be used as a readout for autophagic flux in the Pho8∆60 assay.

The cytosolic phosphatase Pho13 can also turn over para-nitrophenyl phosphate independently of autophagic flux [34]. To decrease this background signal, Pho13 can be genetically deleted. This becomes especially important when attempting to detect small bulk autophagy fluxes. Compared to Pho8, the activity of Pho13 on α-naphthyl phosphate is low. Thus, deletion of *PHO13* is not required when using α-naphthyl phosphate as phosphatase substrate [36,37]. 

*Pho8∆60* can be stably integrated into the genome, replacing the *PHO8* gene [34]. Alternatively, plasmid-based overexpression of Pho8∆60 without deleting endogenous *PHO8* has been used to measure autophagic flux [38,39]. This plasmid-based approach requires continuous selection for the plasmid, but avoids the assay-specific manipulation of the yeast genome, especially when using α-naphthyl phosphate as phosphatase substrate. 

To induce bulk autophagy experimentally, mid-log phase Pho8∆60 yeast is shifted to nitrogen starvation medium [34] or treated with rapamycin [15]. In our hands, nitrogen starvation yielded more reproducible results in the BY474x strain than rapamycin treatment. In any case, bulk autophagy should be induced for more than two hours to ensure that enough active Pho8∆60 is accumulated in the vacuole to obtain a quantitative readout [34]. 

#### 3.1.2. GFP-Atg8 Assays

**Figure 3:**
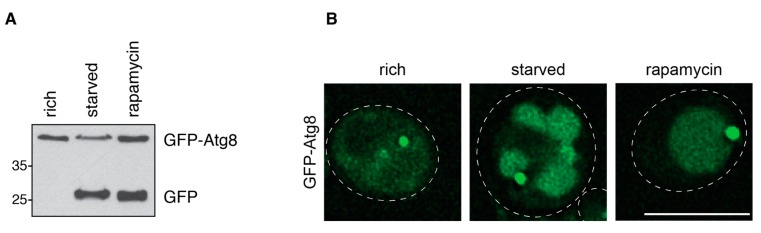
GFP-Atg8 assays to assess bulk autophagic activity. (**A**) Vacuolar delivery of GFP-Atg8 by bulk autophagy assessed by Western blotting using an anti-GFP antibody. The GFP moiety is resistant to vacuolar hydrolases, while Atg8 is degraded. This leads to the appearance of a free GFP band. Cells were grown to mid-log phase overnight (rich) and then nitrogen-starved or rapamycin-treated for six hours. Samples were prepared by TCA (trichloroacetic acid) precipitation; (**B**) Vacuolar delivery of GFP-Atg8 by bulk autophagy visualized by fluorescence microscopy. Cells were grown to mid-log phase overnight (rich) and then nitrogen-starved or rapamycin-treated for six hours. Vacuoles are stained intensely with GFP after starvation and treatment with rapamycin. Note that during starvation, but not during rapamycin treatment, vacuoles appear fragmented in BY474x strains. Due to the upregulation of Atg8 expression during bulk autophagy induction, contrast was adjusted individually for the “rich” image. Dashed lines: cell outlines. Scale bar: 5 µm.

Atg8 is C-terminally processed by the protease Atg4 to expose a glycine residue. Via this residue, Atg8 is then conjugated to the lipid phosphatidylethanolamine (PE) on both sides of the isolation membrane [40,41,42]. While it is removed again from the outer membrane by the action of Atg4, it remains conjugated to the inner membrane of the autophagosome. Thus, Atg8 is delivered to the vacuole with the inner vesicle and degraded by the resident hydrolases [40,43]. To monitor the delivery of Atg8 to the vacuole, Atg8 can be N-terminally tagged with GFP. A C-terminal GFP tag would be lost due to the action of Atg4 on Atg8 [41]. Expressing GFP-Atg8 from the genomic *ATG8* locus or from a CEN plasmid under the control of its endogenous promoter allows assessment of autophagic flux by two different approaches: first, the vacuolar delivery of GFP-Atg8 can be monitored by Western blotting. As GFP is more resistant to the vacuolar degradation than Atg8, bulk autophagy results in the accumulation of free GFP in the vacuole. GFP-Atg8 and free GFP are detectable by Western blotting using a GFP-specific antibody (Figure 3A) [44]. The ratio between both bands can be quantified and used as a readout for the autophagic flux. Second, the GFP signal in the vacuole can be visualized in fluorescence microscopy (Figure 3B) [19]. This visualization requires the delivery of sizable amounts of GFP-Atg8 to the vacuole because the GFP signal is partially quenched in the acidic environment of the vacuolar lumen. Quenching can be avoided by the usage of pH-tdGFP, a recently engineered pH-stable GFP variant [45], or other fluorescent tags such as mCherry [46]. While mCherry offers excellent pH stability, it is significantly less bright than GFP variants [47]. To increase the accumulation of fluorescence signal, vacuolar protein degradation can be blocked chemically by treatment with protease inhibitors such as PMSF (phenylmethanesulfonyl fluoride) or genetically by deleting Pep4, which is required for the activation of other vacuolar proteases [33]. As the fluorescence-based assay measures the delivery of Atg8 to the vacuole, it does not depend on functional vacuolar degradation. If desired, the vacuole can be counterstained with dyes such as FM4-64 [48] or vacuolar proteins tagged with fluorescent proteins spectrally separable from GFP.

Assessing the vacuolar delivery of GFP-Atg8 either by fluorescence microscopy or Western blotting is mainly used to monitor bulk autophagy. Cvt vesicles formed in nutrient-rich conditions also contain Atg8 [43]. Because these vesicles are smaller and formed less frequently, they transport less Atg8 to the vacuole [4]. Thus, the amount of GFP-Atg8 delivered to the vacuole by the Cvt pathway is generally below the detection limit for both readouts. 

The detection of vacuolar GFP can also be applied to determine whether other GFP-tagged proteins are transported to the vacuole via autophagy. These can be either autophagic cargo proteins, cytoplasmic proteins or part of the autophagic machinery (see below). In the last case, this method allows determining whether certain parts of the machinery are excluded from the forming autophagosome or degraded by autophagy. Atg1-GFP and Atg13-GFP, for instance, have been found to be degraded by bulk autophagy [49].

#### 3.1.3. Comparison of the Pho8∆60 and the GFP-Atg8 Assays

The Pho8∆60 assay and the GFP-Atg8 assays are the most commonly used methods to monitor bulk autophagy in yeast. Each has specific strengths and limitations.

The strength of the Pho8∆60 assay is its sensitivity and clear, quantitative readout. It also specifically measures bulk autophagy, with no contribution of selective autophagy pathways. This is because in selective autophagy the isolation membrane specifically enwraps a cargo, excluding other cytoplasmic material such as Pho8∆60. One limitation of the Pho8∆60 assay is an unavoidable background signal even without the induction of bulk autophagy. This background signal makes detecting very low levels of bulk autophagy difficult. If *pho8∆60* needs to be stably integrated in the genome or if *PHO13* needs to be deleted to reduce background, strains must be generated beforehand. This may be avoided by plasmid-based overexpression of Pho8∆60 and the use of α-naphthyl phosphate as phosphatase substrate. However, the Pho8∆60 assay requires a more laborious cell lysis protocol than the GFP-Atg8 assays. This limits its applicability for quick screening experiments.

In contrast, the strength of the GFP-Atg8 assays lies in fast and hassle-free experiments. GFP-Atg8 can be expressed from a plasmid, even on top of endogenous Atg8, avoiding the need for genetic manipulation. In addition, GFP-Atg8 assays allow easily corroborating results by analyzing bulk autophagy with two approaches: Western blotting measuring vacuolar degradation, and fluorescence microscopy monitoring vacuolar delivery. The drawbacks are the high detection limit in fluorescence microscopy due to GFP quenching in the vacuole and the only semi-quantitative readout when assessing GFP-Atg8 cleavage by Western blotting. In the latter case, it is important to ensure that all the bands are within the linear range when quantifying. Atg8 is also conjugated to the isolation membrane in selective autophagy. Thus, selective autophagy pathways also contribute to the vacuolar delivery of Atg8.

In summary, the Pho8∆60 assay is a quantitative and more reliable method to measure bulk autophagy. The GFP-Atg8 assays are the methods of choice to assess bulk autophagy function by two different readouts in a fast and easy way. 

#### 3.1.4. Cytoplasmic Proteins Tagged with GFP

Similar to GFP-Atg8, cytoplasmic proteins can be tagged with GFP to monitor their delivery to the vacuole either by fluorescence microscopy or by the detection of free GFP by Western blotting. GFP-tagged proteins used previously for studying autophagy in this fashion include the highly abundant proteins Pgk1 and Fba1, and the lowly abundant protein Hog1 [50,51]. As these proteins reside in the cytoplasm, their transport to the vacuole solely depends on bulk autophagy. The method shares with the Pho8∆60 assay the advantage of being specific for bulk autophagy. As cytoplasmic material is excluded in selective autophagy, only bulk autophagy contributes to the detected signal. As in the GFP-Atg8 assays, no specific genetic background is necessary, allowing fast and easy testing of bulk autophagy. The limitations of the method are the same as of the GFP-Atg8 assays: the semi-quantitative readout by Western blotting and the high detection limit in fluorescence microscopy due to GFP quenching in the vacuole.

#### 3.1.5. Protein Degradation upon Starvation

Cytoplasmic proteins delivered to the vacuole by bulk autophagy are degraded by resident proteases. The concomitant release of TCA-soluble amino acids can be measured in radioactive pulse-chase experiments and was used as a readout for bulk autophagy in the first autophagy studies in yeast [9]. The cells are labeled with [^14^C] leucine, followed by a chase period in nutrient-rich medium and induction of bulk autophagy. Protein degradation can be measured by assessing the appearance of short radio-labeled peptides which are soluble in TCA. Alternatively, protocols for determining intracellular amino acid amounts without the need for radiolabeling exist [52]. All such assays use the degradation of native cytoplasmic proteins as a readout instead of measuring the vacuolar processing of a single protein.

3.1.6. pH Sensitive Fluorescent Reporter

Another fluorescence microscopy-based assay uses the fluorescent reporter Rosella. Rosella is composed of the relatively pH-stable red fluorescent protein DsRed, and the pH-sensitive green fluorescent protein pHluorin. Rosella remains in the cytoplasm and is absent from the vacuole under nutrient-rich conditions. In the cytoplasm, at neutral pH, both pHluorin and DsRed are fluorescent, and Rosella emits green and red signal. After induction of bulk autophagy, Rosella is delivered to the vacuole in autophagosomes. Due to the low vacuolar pH, pHluorin but not DsRed fluorescence is quenched. This change in the ratio of DsRed to pHluorin fluorescence upon vacuolar delivery allows to clearly distinguish cytosolic from vacuolar Rosella without counterstaining the vacuole [53].

### 3.2. End-Point Measurements to Monitor Selective Autophagy

In selective autophagy, certain organelles and cellular components are specifically targeted by the autophagic machinery, and selectively and exclusively engulfed by an isolation membrane [4,5]. These components—the cargo—are recognized by cargo-specific receptor proteins, which bind the selective autophagy scaffold protein Atg11 [3]. Atg11 then links the cargo to the core autophagic machinery [54]. 

**Figure 4:**
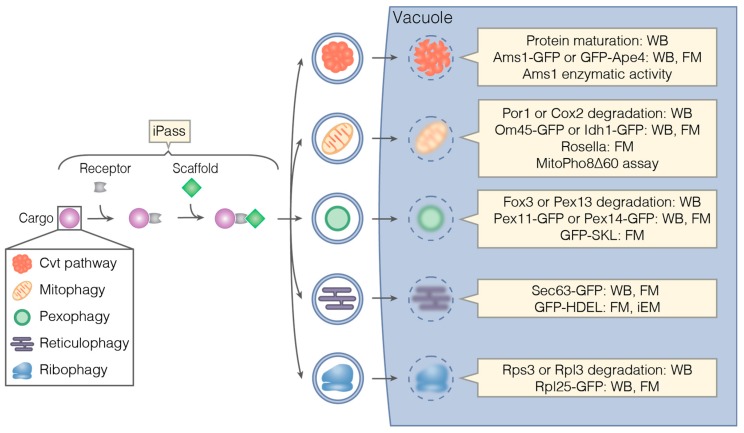
Methods for studying different types of selective autophagy. iPass is a versatile tool to investigate early events in selective autophagy, but can be applied to many other questions as well—see Figure 6B for details. Many methods to study selective autophagy are based on degradation of cargo-specific proteins, vacuolar delivery of fluorescently tagged cargo structures, enzymatic assays or electron microscopy—see text for details. While ribophagy is a selective autophagy pathway, no cargo receptor for ribosomes has been identified yet. FM: fluorescence microscopy; iEM: immuno-electron microscopy; WB: Western blotting.

Depending on the cargo, selective autophagy is classified into different types (Figure 4). Pathway-specific methods and their application are described in individual sections below. However, several end-point assays can be applied to more than one type of selective autophagy. First, the degradation of a specific selective autophagy cargo can be monitored by Western blotting using an antibody recognizing an endogenous protein that is part of the cargo. Second, the vacuolar delivery of the cargo can be tested by isolating vacuoles and probing for the presence of the cargo in the vacuolar fraction. To ensure that the cargo is not merely associated with the vacuole, its luminal localization can be tested with a protease protection assay. Treatment of the isolated vacuoles with proteinase K leads to the degradation of vacuole-associated proteins. Cargo inside the vacuolar lumen is protected from protease treatment by the vacuolar membrane [23]. Third, similar to the GFP-Atg8 assays, the vacuolar delivery of a cargo-specific protein tagged with GFP can be monitored by either fluorescence microscopy or Western blotting. Fourth, the fluorescent reporter Rosella [53], described for bulk autophagy above, can be targeted to a specific cargo. The vacuolar delivery can then be monitored by fluorescence microscopy. Fifth, Pho8∆60, described for bulk autophagy above, can be targeted to a cargo organelle by fusion to a cargo protein or signal sequence. The vacuolar delivery of the cargo can be measured as increased phosphatase activity [55]. Sixth, the content of autophagic vesicles can be directly visualized by electron microscopy, which allows directly assessing whether a certain type of cargo is degraded by selective autophagy. An overview of assays to monitor different types of selective is provided in Figure 4. 

To increase the signal in many of the assays, the proliferation of the targeted organelle of interest can be enhanced before the induction of selective autophagy. Conditions that trigger proliferation vary between organelles and are detailed in the respective sections below.

Selective autophagy of organelles often needs to be induced by nitrogen starvation [3], which also leads to the activation of bulk autophagy. In such cases, it is imperative to ensure that the cargo of interest is degraded selectively rather than randomly by bulk autophagy. One hallmark of the degradation by selective autophagy is that it progresses faster than the random degradation of cytoplasmic components, which can be tested by monitoring general protein degradation. In addition, most selective autophagy pathways depend on a cargo receptor and the selective autophagy scaffold protein Atg11 [3]. This dependence can be tested for by deletion of either Atg11 or the cargo receptor, if it is known. 

#### 3.2.1. The Cvt Pathway

**Figure 5:**
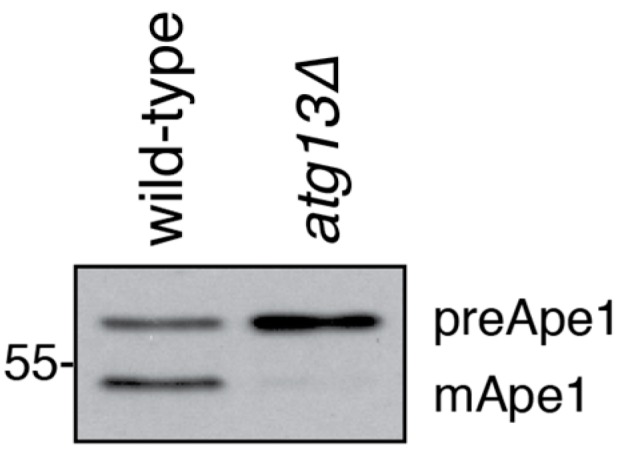
Ape1 processing as a readout for Cvt pathway activity. The Cvt pathway delivers the inactive precursor of Ape1 (preApe1) to the vacuole. There, preApe1 is proteolytically processed to its mature, active form (mApe1). The resulting shift in size can be detected by Western blotting using an Ape1-specific antibody. In Cvt-deficient mutants such as *atg13∆*, Ape1 maturation is decreased or absent. Cells were grown to mid-log phase overnight. Samples were prepared by TCA precipitation.

The Cvt pathway delivers the cytosolic proteins Ape1, Ams1 and Ape4 to the vacuole. The vacuolar transport of Ape1 is widely used in the field as a model pathway for selective autophagy. The inactive precursor of Ape1 (preApe1) is recognized by the cargo receptor Atg19 and engulfed by an isolation membrane forming a so-called Cvt vesicle [4,56]. Once delivered to the vacuole, the N-terminal propeptide of preApe1 is proteolytically cleaved, yielding the enzymatically active, mature form mApe1. The maturation of Ape1 causes a change in molecular weight, which can be detected by Western blotting with an antibody binding to the C-terminus of Ape1, thus recognizing both preApe1 and mApe1 (Figure 5) [13,21]. The ratio of the mApe1 to the preApe1 band is a simple and sensitive readout of Cvt activity, and as such is widely used in the field. However, similar to other approaches based on Western blotting, it is only semi-quantitative due to the inherent heterogeneity of Western blotting. Radioactive pulse-chase experiments offer an alternative and more quantitative method that is not based on Western blotting. Radioactive labeling followed by a non-radioactive chase and immunoprecipitation of Ape1 for different time periods allows to monitor the kinetics of the Cvt pathway [21]. 

The Cvt pathway is constitutively active in nutrient-rich conditions. Its activity is enhanced when bulk autophagy is induced experimentally or when cells reach stationary phase, but remains selective and dependent on Atg11 and Atg19 in these conditions. mApe1 is resistant to vacuolar proteases and accumulates in the vacuole when the Cvt pathway is upregulated [57]. mApe1 accumulated during stationary phase is stable for several hours also after cells are shifted to nutrient-rich medium. This may strongly influence experimental results when studying constitutive Cvt activity in nutrient-rich conditions. To ensure that excessively accumulated mApe1 is degraded before assessing Cvt activity, cells need to be grown in logarithmic phase overnight before being harvested in mid-log phase.

Besides Ape1, the Cvt pathway also transports Ams1 and Ape4 from the cytoplasm to the vacuole [22,23]. Ams1 is recognized by two partially redundant cargo receptors, Atg19 and Atg34 [56,58]. The vacuolar transport of Ape4 also depends on Atg19 [23]. Both Ams1 and Ape4 are cleaved in the vacuole. This cleavage can be detected using antibodies specific for either protein on Western blots. In the case of Ams1, obtaining a robust signal of the cleaved product has been reported to require Ams1 overexpression and cultivation of cells in a non-fermentable medium [22]. To avoid protein overexpression, the transport of Ams1-GFP to the vacuole can be monitored by detecting free GFP on a Western blot or by fluorescence microscopy as in the GFP-Atg8 assays described earlier [58]. Alternatively, Ams1 activity can be determined by an enzymatic assay on isolated vacuoles [22]. As for Ams1, transport of Ape4 to the vacuole can be monitored by detecting either vacuolar processing of the endogenous protein with an Ape4-specific antibody or the formation of free GFP in the vacuoles of cells expressing GFP-Ape4 [23]. Both approaches are impeded by the low amounts of Ape4 constitutively transported to the vacuole by the Cvt pathway. As Ape4 transport is considerably upregulated upon starvation, the obtained signal can be increased by shifting cells to starvation medium. Although bulk autophagy is induced under these conditions, the majority of Ape4 transport occurs selectively and depends on both Atg11 and Atg19 [23]. This dependence needs to be tested for in any experiments using starvation to boost Ape4 transport.

#### 3.2.2. Mitophagy

Mitochondria are important producers of ATP, especially during respiratory growth on non-fermentable carbon sources such as lactate. In such conditions, mitochondria biogenesis is upregulated. The additional mitochondria become superfluous in the presence of fermentable carbon sources such as glucose and are degraded by a selective autophagy pathway called mitophagy. Mitophagy is mediated by the cargo receptor Atg32, which binds to Atg11 [59,60]. Mitochondria are then enwrapped in an isolation membrane and delivered to the vacuole. 

Mitophagy can be assessed by monitoring the vacuolar degradation of mitochondrial proteins. This degradation has been analyzed by Western blotting with antibodies against the mitochondrial outer membrane protein Por1 or the mitochondrial inner membrane protein Cox2 [61]. Similar to the bulk autophagy GFP-Atg8 assays, mitophagy can be analyzed by monitoring the transport of GFP-tagged mitochondrial proteins to the vacuole by fluorescence microscopy or Western blotting. Om45, a mitochondrial outer membrane protein, and the matrix protein Idh1 have been tagged with GFP and employed in this manner [62]. To fluorescently label mitochondria, fluorescent proteins can also be fused to the targeting signal of mitochondrial proteins such as Cit2 or Cox4. The DsRed/pHluorin dual color fluorescent reporter Rosella has been targeted to mitochondria this way [53]. Cytoplasmic Rosella is used to analyze bulk autophagy (see above); however, when targeted to mitochondria it can be employed to monitor mitophagy. When Rosella-containing mitochondria are delivered to the vacuole by mitophagy, the low pH in the vacuole quenches the pHluorin emission, while DsRed remains fluorescent. The detection of DsRed signal in the absence of pHluorin signal is a sensitive readout for the vacuolar delivery of mitochondria. In principle, Rosella can also be targeted to other intracellular structures to analyze their delivery to the vacuole.

Another way to monitor mitophagy utilizes an adapted form of the Pho8∆60 assay described for bulk autophagy. In the mitoPho8∆60 assay, Pho8∆60 is targeted to mitochondria by fusing it to the mitochondrial targeting signal of Cox4 [55]. The induction of mitophagy leads to the transport of mitoPho8∆60 to the vacuole, where it is activated. As for the cytoplasmic Pho8∆60 assay, the clear and quantitative readout of the mitoPho8∆60 assay is an advantage over GFP cleavage assays. The principle of organelle-specific Pho8∆60 assays is in theory applicable to other organelles as well. 

Mitophagy is induced when cells are shifted from nutrient-rich to nitrogen starvation medium, both containing a fermentable carbon source such as glucose [63,64]. To increase the signal in mitophagy assays, mitochondria proliferation can be promoted by pre-cultivating cells on non-fermentable carbon sources such as lactate or glycerol. To induce mitophagy, cells can then either be grown to stationary phase in the same medium or can be nitrogen-starved in the presence of glucose [61,62,65]. Although bulk autophagy is triggered in these conditions, mitophagy remains a selective process dependent on its cargo receptor Atg32 and Atg11 [59,62]. These dependencies need to be controlled for in any experiments in which bulk autophagy is induced.

In contrast to mammalian cells, treatment with the mitochondrial uncoupler CCCP (carbonyl cyanide m-chlorophenylhydrazone) does not induce mitophagy in yeast [61]. However, mitochondrial damage in general might trigger mitophagy. Damage to mitochondria caused by the depletion of Mdm38 has been reported to induce their delivery to the vacuole [66].

#### 3.2.3. Pexophagy

Peroxisomes are specialized organelles carrying out metabolic processes such as the catabolism of certain fatty acids by β-oxidation. Cells regulate the number of peroxisomes to adapt to changing nutrient conditions. Growth on certain carbon sources, such as long-chained fatty acids, requires high peroxisomal activity and triggers peroxisome proliferation [67]. In the presence of a preferred carbon source such as glucose, or when nutrients are generally lacking, the additional peroxisomes become superfluous, and are degraded by selective autophagy, a process called pexophagy [68]. Upon induction of pexophagy, the pexophagy cargo receptor Atg36 links Atg11 to Pex3 on peroxisomes [69]. Peroxisomes are then sequestered by an isolation membrane and delivered to the vacuole. 

Pexophagy can be investigated by monitoring the vacuolar degradation of endogenous peroxisomal proteins. Western blotting for Pot1/Fox3, a peroxisomal 3-ketoacyl-CoA thiolase [70,71], and Pex13 [72] have been used in this manner previously. Alternatively, peroxisomal proteins such as Pex11 or Pex14 can be tagged with GFP to monitor delivery of peroxisomes to the vacuole by fluorescence microscopy or GFP cleavage assays [69,73]. For fluorescence microscopy experiments, GFP can also be targeted directly to peroxisomes via the type I peroxisomal targeting signal serine-lysine-leucine (GFP-SKL) [74].

To increase the signal in pexophagy assays, the proliferation of peroxisomes can be triggered by changing the carbon source before the induction of pexophagy. Many studies on peroxisomes have been conducted in the methylotrophic yeasts *Pichia pastoris* and *Hansenula polymorpha*, which are able to metabolize methanol depending on peroxisomal function [75]. These yeasts strongly proliferate peroxisomes when cultivated on methanol as the sole carbon source. Pexophagy then is induced by simply shifting the cells to a medium containing glucose or ethanol. In *Saccharomyces cerevisiae*, proliferation of peroxisomes is triggered when cells are cultivated on oleic acid, which is metabolized by β-oxidation in peroxisomes [67]. Pexophagy has been induced by simply shifting *S. cerevisiae* from oleic acid to glucose-containing medium [70]; however, pexophagic flux is commonly increased by simultaneous nitrogen starvation [71]. Even without triggering peroxisome proliferation by growth on oleic acid, pexophagy can be induced by shifting cells from glucose-containing, nutrient-rich medium to nitrogen starvation medium [72]. Even in starvation conditions, peroxisomal degradation is a selective process dependent on the cargo receptor Atg36. This dependency needs to be controlled for in any experiments in which bulk autophagy is induced to accelerate pexophagy.

#### 3.2.4. Reticulophagy

The endoplasmic reticulum (ER) is a central hub for protein folding, sorting and secretion. The degradation of portions of the ER via selective autophagy is called reticulophagy. Reticulophagy depends on two cargo receptor proteins, Atg39 and Atg40, which link the ER to the autophagic machinery via Atg11 [76]. 

To tag the ER for reticulophagy studies, fluorescent proteins can be fused to ER proteins such as Sec63 or directly targeted to the ER by the ER retrieval signal histidine-aspartate-glutamate-leucine (HDEL). This allows assessing reticulophagy by fluorescence microscopy, immuno-electron microscopy or by performing fluorescent protein cleavage assays [76,77]. 

Reticulophagy can be induced by nitrogen starvation or rapamycin treatment [76,77]. Although these conditions trigger bulk autophagy, reticulophagy proceeds in a selective manner dependent on the cargo receptors Atg39 and Atg40, and their binding to Atg11 [76]. These dependencies need to be controlled for in any experiments in which bulk autophagy is induced to trigger reticulophagy. 

Engulfment of portions of the ER in double membrane-bounded structures reminiscent of autophagosomes has also be observed after long-term induction of ER stress by treatment with DTT (dithiothreitol) [78]. However, these structures have not been found to fuse with the vacuole. They seem to serve ER sequestration rather than degradation and may not represent canonical reticulophagy.

#### 3.2.5. Ribophagy

Ribosomes are highly abundant, complex macromolecular machines responsible for protein synthesis. In starvation conditions, when protein synthesis is downregulated profoundly, a large number of ribosomes become superfluous. These are degraded by a selective autophagy pathway referred to as ribophagy [51]. In addition to selective ribophagy, also bulk autophagy randomly degrades ribosomes due to their abundancy and small size.

To study ribophagy, the degradation of the ribosomal proteins Rps3 and Rpl3 can be monitored by Western blotting. In addition, delivery of GFP-tagged ribosomal proteins such as Rpl25-GFP to the vacuole can be assessed either by fluorescence microscopy or GFP cleavage assays, similar to the bulk autophagy GFP-Atg8 assays.

Ribophagy is induced by shifting cells to nitrogen starvation medium. These conditions also induce bulk autophagy. To determine whether the ribosomes are delivered to the vacuole via bulk autophagy or selective ribophagy, a cytoplasmic protein such as Hog1 should be used as a control for random degradation by bulk autophagy [51]. A faster turnover of ribosomes compared to the cytoplasmic control protein indicates that ribosomes are degraded selectively in addition to non-specific turnover via bulk autophagy. 

## 4. Monitoring Individual Steps during Autophagy

### 4.1. Autophagic Machinery Assembly

In yeast, autophagosomes are formed at the vacuolar membrane at the PAS [19]. Rather than being a constitutive structure, the PAS is defined by the co-localization of Atg proteins visible as perivacuolar dot in fluorescence microscopy. This co-localization of proteins is one of the earliest steps in the autophagy pathway. It is a hierarchical process starting from Atg17 in bulk autophagy, and from the cargo in selective autophagy [54,79]. 

Fluorescence microscopy can be used to study the assembly of the autophagic machinery at the PAS or the recruitment of Atg proteins to the cargo, indicating whether the first steps of autophagy are functional [19,54]. To do so, the co-localization of one or several proteins of interest with a PAS marker protein or the cargo is assessed. A PAS marker should form a single, bright, perivacuolar dot in fluorescence microscopy and ideally be recruited to the PAS at an early stage. In bulk autophagy, several core autophagy proteins are suitable PAS markers, from which Atg17 stands out as the most upstream protein in PAS assembly. Atg8 is commonly used as a PAS marker but is thought to be recruited downstream of several other proteins during PAS formation [79]. As such, Atg8 may not be the preferred marker when studying early steps in PAS assembly. Atg9, and its accessory proteins Atg23 and Atg27, localize to numerous, highly mobile vesicles in the cytoplasm in addition to the PAS [80,81,82,83,84], and thus are not suitable as PAS markers. 

To better analyze autophagy machinery formation in fluorescence microscopy, Atg proteins can be accumulated at the PAS by stalling autophagosome formation. This is achieved by deleting proteins such as Atg1 or Atg2. In the absence of these proteins, the early autophagy machinery still assembles at the PAS, but no autophagosomes can be completed [79,82,85]. The transport-of-Atg9-after-knocking-out-*ATG1* (TAKA) assay is based on this principle [86]. It is used to study the effect of mutations on the recruitment of Atg9 to the PAS. Knocking out Atg1 on top of the mutation of interest blocks the release of Atg9 from, but not its recruitment to the PAS. Thus, the TAKA assay allows differentiating between defects in the recruitment of Atg9 vesicles to the PAS and an increase of Atg9 retrograde transport away from the PAS.

The protein stoichiometry at the PAS can be quantified by correlating fluorescence intensities to protein amounts determined by Western blotting [87]. To avoid estimating the protein amount by Western blotting, a protein such as Cse4-GFP forming punctate structures of known stoichiometry can be used as an intensity standard in fluorescence microscopy [84].

### 4.2. Protein Recruitment to Cellular Structures

**Figure 6:**
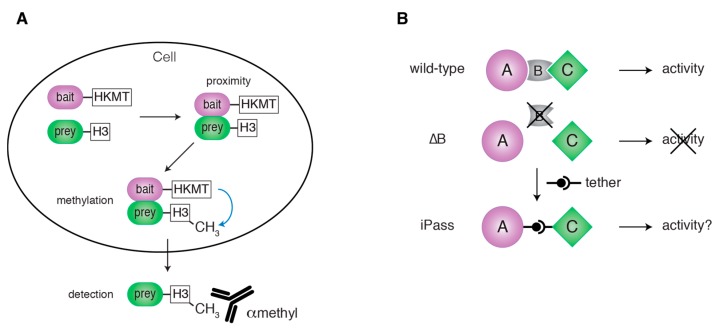
In vivo assays to monitor individual steps during a signaling pathway. (**A**) The protein proximity assay M-Track. A bait protein is tagged with a histone lysine methyltransferase (HKMT) and a prey protein with a fragment of histone H3 (H3). Close proximity between bait and prey leads to the methylation of the H3 tag by HKMT. This methylation mark is detected by Western blotting using an antibody specific for H3 lysine methylation. (**B**) iPass (induced bypass). When the bridging factor B of a trimeric complex (A-B-C) is deleted, the function of the complex is lost. In iPass, A and C are inducibly tethered in the absence of B. iPass restores the activity of the complex if the only non-redundant role of B is to link A and C. iPass can be used to study the function of B or to temporally control the progression of the pathway governed by the complex A-B-C—see text for details.

Fluorescence microscopy is a powerful tool to detect proteins concentrated on cellular structures such as organelle membranes or the PAS. However, the signal from low amounts of protein localizing to a structure of interest may be masked by the signal from the cytoplasmic protein pool. Protein proximity assays offer a sensitive way to overcome this limitation. BioID, a biotinylation-based protein proximity assay, has been successfully applied in mammals [88]. In yeast, strong endogenous protein biotinylation limits the applicability of BioID. The methylation-based M-Track assay, on the other hand, is uniquely suited for detecting protein proximity in yeast [89,90]. In M-Track, two proteins of interest are tagged with a human histone 3 lysine 9 methyltransferase (HKMT) and several copies of a fragment of histone H3 (H3), respectively. If the two proteins are in close proximity, lysine 9 in the H3 fragment (H3K9) will be methylated. This methylation event can be detected by Western blotting with an antibody recognizing H3K9 methylation (Figure 6A). As yeast lacks endogenous enzymes for H3K9 methylation and demethylation, the methylation mark is specific for protein proximity and persists permanently. This makes M-Track an extremely sensitive and essentially background-free assay. Even though M-Track is a protein proximity assay, it has been used to detect short-lived protein-protein interactions [20,89]. In contrast to classical interaction assays such as co-IP, M-Track alone does not allow to differentiate between close proximity and actual interaction. However, the M-Track methylation mark is applied quickly and specifically in living cells, and is not subject to lysis or post-lysis artifacts. This makes M-Track a versatile tool also when studying protein-protein interactions. Our group recently applied M-Track to show that Atg1 localizes to the vacuolar membrane in nutrient-rich conditions independently of cargo [72]. The pool of Atg1 localizing to the vacuolar membrane was too small to be detected by fluorescence microscopy. M-Track, however, easily captured the proximity of Atg1 to vacuolar membrane proteins.

Alternatively, protein recruitment to cellular structures can be determined by performing cell fractionation assays [40]. Cell fractionation allows screening many cellular structures for the localization of one or several proteins of interest in a single experiment. However, the specificity of the results strongly depends on the purity of the cellular fractions obtained. 

### 4.3. Bypassing Protein Functions in Distinct Steps of Autophagy

Historically, protein functions have been determined by observing the phenotype caused by genetic deletion of the protein of interest. However, the observed phenotype does not necessarily reveal the specific molecular function directly. In addition, if the deletion causes a block of a pathway at an early stage, this approach will fail to capture potential additional roles of the protein in later steps. To overcome these limitations, our group developed iPass (induced bypass), a synthetic in vivo approach which combines genetic deletion with induced dimerization (Figure 6B) [72]. iPass can be applied to study non-redundant functions of proteins involved in the formation of protein complexes or the recruitment of binding partners to cellular structures. iPass attempts to bypass the function of a bridging factor in a protein complex. The bridging factor is deleted, blocking assembly of the complex and thus abrogating pathway function. The other complex members are then brought together synthetically, e.g., by chemically-induced dimerization. If this restores pathway progression, the only non-redundant function of the protein of interest is bridging the other complex members. If not, it likely serves additional roles in the same or a later stage of the pathway. As outlined above, such roles later in the pathway cannot be captured in classical genetic deletion studies, but may be studied in detail in the iPass situation.

### 4.4. Atg13 Phosphorylation

**Figure 7:**
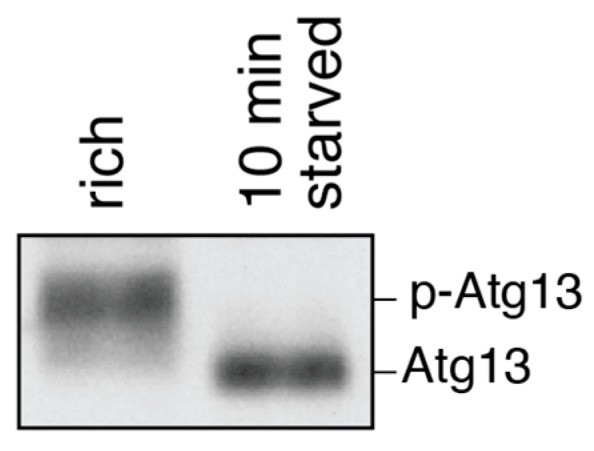
Atg13 is rapidly dephosphorylated upon starvation. Atg13 is highly phosphorylated (p-Atg13) under nutrient-rich conditions, but becomes dephosphorylated within minutes of nitrogen starvation. Cells were grown to mid-log phase (rich) and then nitrogen-starved for 10 min. Samples were prepared by TCA precipitation. Atg13 phosphorylation was determined by Western blotting using an Atg13-specific antibody. Note that complete dephosphorylation of Atg13 has been reported to occur within one minute of starvation [94].

Atg13 is phosphorylated by TORC1 and PKA in nutrient-rich conditions, but is dephosphorylated within minutes of starvation or rapamycin treatment [91,92,93,94]. Thus, the phosphorylation state of Atg13 serves as a readout for the functionality of upstream signaling cascades inducing or inhibiting bulk autophagy. In addition, it can be used to determine whether cells were starved during cultivation or harvesting. Atg13 phosphorylation can be monitored by Western blotting using antibodies against Atg13 or against recombinant epitope tags such as HA [91,93]. On such a blot, phosphorylated Atg13 is visible as a smear, which collapses to a sharp band when Atg13 is dephosphorylated (Figure 7).

### 4.5. Atg1 Kinase Activation

Like many other serine/threonine kinases, Atg1 autophosphorylates at least two sites within its activation loop, likely in trans. This autophosphorylation is necessary for the kinase activity both during selective and bulk autophagy [95,96,97]. Basal Atg1 activity can be detected also in nutrient-rich conditions, but kinase activity is increased upon starvation about 5–7 fold [72,91]. Currently, it is unknown whether the observed increase in activity comes from a larger pool of activated Atg1, from individual Atg1 molecules being more active, or both. 

When studying Atg1 activity in nutrient-rich conditions, special care is required when harvesting cultures. Atg1 activity strongly depends on the phosphorylation state of its binding partner Atg13 [91]. Atg13 becomes dephosphorylated rapidly, increasing Atg1 activity when cells are stressed or starved, both of which may happen during harvesting by centrifugation [98]. To preserve the nutrient-rich state, cells should be harvested rapidly by filtration and immediately frozen in liquid nitrogen [72]. Filtration offers a simple, fast and mild way to harvest cells without inducing the starvation response. Frozen cells can be lysed directly by cryogenic grinding. This way, cells and cell lysate are continuously frozen, minimizing the risk of changes in autophagic signaling during harvest or lysis [72]. To preserve the nutrient-rich phosphorylation state of Atg13, it is imperative to keep cell lysates at 4 °C and add sufficient amounts of phosphatase inhibitors [72,98]. A simple way to control whether the nutrient-rich state is preserved is to check Atg13 phosphorylation as described above. 

In vitro phosphorylation assays offer a sensitive way to measure Atg1 kinase activity. Atg1 complexes are immunoprecipitated from yeast lysate prepared by cryogenic grinding and then mixed with radioactively labeled ATP and a suitable substrate for Atg1 [49,91]. Atg1 autophosphorylation or substrate phosphorylation can be used as a readout for the kinase activity. Myelin basic protein (MBP) [91], the C-terminus of Atg9 [20] and the C-terminus of Atg19 [99] have been used as Atg1 substrates previously.

In nutrient-rich conditions, the autophagic machinery including Atg1 assembles on the Cvt pathway cargo, preApe1. When studying Atg1 kinase activity in these conditions, care needs to be taken in order not to lose preApe1 complexes with membrane debris when clearing the cell lysate by centrifugation prior to immunoprecipitating Atg1. As Atg1 is activated on preApe1 complexes, loss of these complexes might cause the loss of the most active Atg1 pool [72,100]. 

For qualitatively assessing Atg1 activity, Atg1 autophosphorylation can also be evaluated by Western blotting. Autophosphorylation leads to a size shift or smear of the Atg1 band [91]. This effect can be enhanced by using Phos-tag in the SDS-PAGE [101,102]. The Phos-tag reagent binds to phosphate groups on phosphorylated proteins, increasing the apparent size shift between the phosphorylated and unphosphorylated states. While these Western blotting approaches offer a simple way to test whether Atg1 is active without the need for immunoprecipitation, in vitro phosphorylation assays are the method of choice when quantitatively assessing Atg1 activity.

### 4.6. Atg8 Lipidation

Atg8 is a small ubiquitin-like protein that is conjugated to the lipid PE by an E1-E2-E3-like conjugation system [42]. Its conjugation on the isolation membrane is essential for bulk and selective autophagy [41]. The lipidated form of Atg8 (Atg8-PE) migrates faster than non-lipidated Atg8 in SDS-PAGE with gels containing urea. This shift in size can be detected by Western blotting with an antibody specific for Atg8 [41,42]. The Atg8 lipidation assay probes for the functionality of the Atg8 conjugation machinery. It is not suited for studying general autophagy function, as even in mutants impaired in early steps of autophagy a sizable amount of Atg8 becomes lipidated by its conjugation machinery [38].

### 4.7. Autophagosome Formation

**Figure 8:**
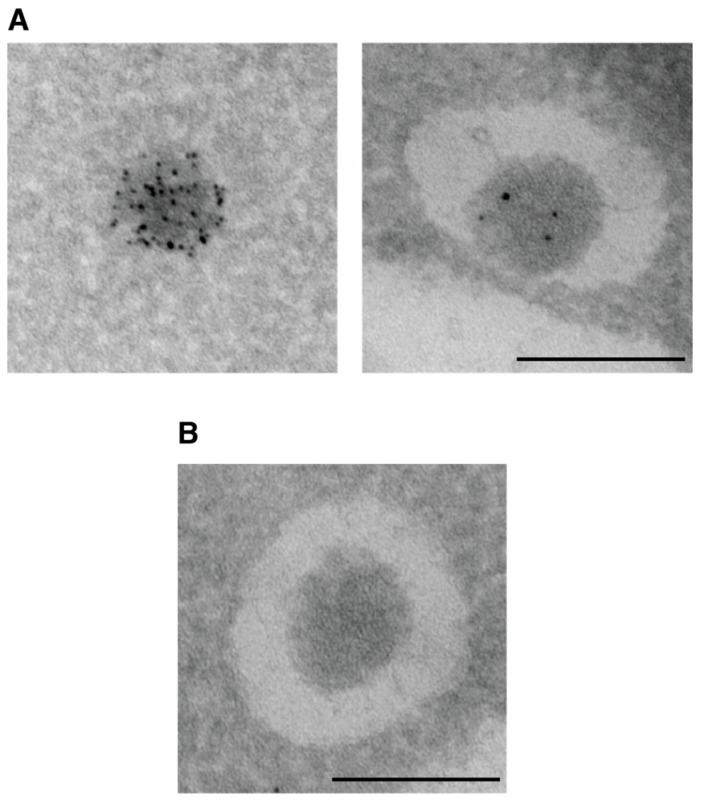
Identification of Cvt vesicles in immuno-electron microscopy. (**A**) Immuno-labeling of Ape1 with gold-coupled antibodies is commonly used to identify Cvt vesicles. Ape1 oligomers before (**left**) and after (**right**) sequestration in a Cvt vesicle are shown. (**B**) The unique ultrastructure of the Ape1 oligomer can be easily recognized even in the absence of gold particles. (**A**,**B**) Due to the low protein content of autophagic membranes, these membranes appear as a white halo-like ring around the Ape1 oligomer. Mid-log phase cells were fixed, embedded and cryo-sectioned by the Tokuyasu method as described in [72]. Ape1 was visualized with an anti-Ape1 antibody recognized by a gold-coupled (6 nm) secondary antibody. Scale bar: 200 nm.

Autophagosomes can be visualized by electron microscopy as double-membrane vesicles with a diameter of 300 to 900 nm [33,103]. Accumulation of completed autophagosomes in the cytoplasm indicates a defect in the autophagosomal-vacuolar fusion. To increase the number of observable autophagosomes experimentally, they can be accumulated in the cytoplasm by deleting the Rab family GTPase Ypt7 or the vacuolar t-SNARE Vam3. Both deletions prevent the fusion of the autophagosome with the vacuole [40,104,105]. Electron microscopy was already used in early studies on autophagy in yeast to detect forming and complete autophagosomes [103]. It can also be employed to distinguish between bulk and selective autophagy. During bulk autophagy, isolation membranes engulf random cytoplasmic material, while in selective autophagy specific cargo is enwrapped selectively and exclusively. Autophagic vesicles containing cytoplasm in addition to structures targeted by selective autophagy are thus likely to be bulk autophagosomes. In addition, Cvt vesicles can easily be identified in electron micrographs due to their smaller diameter of about 150 nm [4]. In electron microscopy, the unique oligomeric structure of the main Cvt cargo, Ape1, is readily recognizable (Figure 8). 

The distribution of Atg proteins on the growing isolation membrane, and on or in the closed autophagosome can be studied by immuno-electron microscopy. A limitation of immuno-electron microscopy is that the sample fixation method must be mild to preserve the epitopes recognized by the antibody [106]. Such mild fixation methods generally do not preserve cell morphology as well as harsher procedures. This is particularly true for the morphology of autophagic membranes, which is hard to preserve by fixation due to the low membrane protein content [107]. These membranes often appear as white, halo-like rings around autophagic cargo rather than as two individual membranes visible by classical fixation (see, e.g., Figure 7C in [106] and Figure 8).

Protease protection assays offer a biochemical approach to test for the completion of autophagosome formation. When autophagosomes are closed, their content becomes protected from treatment with proteinase K. Ape1 and GFP-Atg8 are commonly used as marker proteins for the content Cvt vesicles and bulk autophagosomes, respectively. The propeptide of preApe1 is more sensitive to protease treatment than the rest of the protein. Thus, unprotected preApe1 is processed at its N-terminus when exposed to proteinase K. This shift in size can be detected by Western blotting with an antibody binding to the C-terminal part of Ape1, thus recognizing both full-length and N-terminally processed Ape1 [13,21,108]. Similarly, GFP-Atg8 is processed to free GFP by proteinase K unless protected within an autophagosome [109]. GFP-Atg8 is also conjugated to the inner membrane of Cvt vesicles. However, Cvt vesicles are smaller and fewer in number than autophagosomes, strongly limiting the amount of GFP-Atg8 protected within them. When investigating selective autophagy pathways other than Cvt, the protection of cargo-specific proteins from proteases can be used as a readout for cargo sequestration by autophagic membranes. Only proteins exposed on the cytosolic surface of a cargo are suitable markers in protease protection assays. Proteolytic processing of a marker protein after delivery to the vacuole would confound the results of protease protection assays. To avoid this problem, a fusion-deficient mutant such as *ypt7Δ* can be used in such assays (compare Figure 3C and Figure S2F in [20]).

### 4.8. Vacuolar Delivery and Autophagic Body Degradation

The last steps during autophagy are the fusion of the outer autophagosome membrane with the vacuole, the release of the inner membrane into the vacuolar lumen and the degradation of the autophagic body [33]. 

To determine whether vacuolar delivery is functional, autophagic bodies can be accumulated in the vacuole and counted. To accumulate autophagic bodies, their vacuolar degradation is blocked genetically by deleting the vacuolar protease Pep4, or chemically by treating the cells with the protease inhibitor PMSF [33]. Visualizing the accumulated autophagic bodies by electron microscopy allows not only quantifying their number, but also their size. This is useful to study factors regulating the formation, expansion and completion of autophagosomes. Using this method, Atg8 levels have been found to regulate the size of autophagosomes, while Atg9 levels influence the frequency at which autophagosomes are formed [110,111,112].

To test whether the disassembly of autophagic bodies is functional, autophagic bodies can be quantified in cells without artificially blocking vacuolar degradation as outlined above. In this case, accumulation of autophagic bodies in the vacuole is a readout for an impairment in autophagic body disassembly. This method has been used to identify Atg15, a putative lipase required for the degradation of autophagic bodies in the vacuole [113,114].

As a simple and fast readout, autophagic bodies accumulating in the vacuole can also be detected in bright-field microscopy [33]. Similarly, accumulating autophagic bodies in the vacuole can be visualized in fluorescence microscopy using GFP-Atg8 as a fluorescent marker [114]. As GFP-Atg8 remains conjugated to the inner membrane of autophagosomes, autophagic bodies appear as GFP-positive dots inside the vacuole. Nevertheless, electron microscopy remains the method of choice for studying autophagic bodies in detail, as it allows assessing not only their number, but also their content and size.

### 4.9. Efflux of Catabolites from the Vacuole

After the degradation of cytoplasmic material in the vacuole, the building blocks are exported to the cytoplasm and reused. End-point measurement assays as described above fail to capture defects in catabolite efflux because it occurs downstream of cargo degradation, which serves as the assays’ readout.

Similar to general defects in bulk autophagy, deficiencies in vacuolar efflux cause both decreased viability and decreased protein synthesis in starvation conditions [115,116]. To discern between these two kinds of defects, end-point experiments to measure cargo degradation must be combined with viability and protein synthesis assays. Normal vacuolar degradation of cellular material, but reduced viability and protein synthesis upon starvation, indicate that the efflux of catabolites from the vacuole is defective. Such a phenotype can be observed in cells lacking Atg22 [116]. Directly measuring amino acid abundance in the cytosol may present an intriguing alternative to viability or protein synthesis assays. Protocols for such assays exist [52], but to our best knowledge have not been applied to autophagy research yet. 

### 4.10. Early Steps during the Cvt Pathway

As model for selective autophagy, the Cvt pathway has the advantage over other types of selective autophagy that it can be studied in nutrient-rich conditions, when bulk autophagy is inhibited. However, its constitutive nature is a disadvantage when studying early steps of selective autophagy. Without a way to temporally control the induction of the Cvt pathway, its short-lived early steps are difficult to study. Ape1 processing can be induced by shifting *vac8∆* mutants from nutrient-rich to starvation medium [94,112]. Deletion of Vac8 results in a complete defect of the Cvt pathway in nutrient-rich conditions; however, this defect is rescued when the cells are shifted to starvation medium, providing temporal control over Ape1 processing [94]. As starvation also induces bulk autophagy, the relative contributions of selective and bulk autophagy to the observed processing of Ape1 may be difficult to estimate. To overcome this problem, our lab utilized iPass as described above to induce the Cvt pathway [72]. Tethering the selective autophagy scaffold Atg11 to the Cvt cargo Ape1 in the absence of Atg19 by iPass completely restores Cvt function and allows the induction of Ape1 processing at a desired time also during nutrient-rich growth.

Cvt vesicles are approximately 150 nm in diameter [4] and cannot be resolved by light microscopy. However, when Ape1 is highly overexpressed, it forms an oligomer that is too large to be fully engulfed by the isolation membrane. This so-called giant Ape1 assay allows to monitor the assembly of the autophagic machinery on the Cvt cargo and the formation of the isolation membrane by fluorescence microscopy [117]. Due to the large size of the cargo, isolation membrane elongation requires rapamycin treatment. Like starvation, rapamycin treatment increases the expression of several Atg proteins, which otherwise limits the maximal size of forming autophagic membranes [111]. This defined triggering event allows studying the dynamics of isolation membrane elongation by time-lapse imaging. Despite the requirement for rapamycin treatment, isolation membrane expansion around giant Ape1 complexes is a selective process depending on the cargo receptor Atg19 [99]. 

## 5. Conclusions

*Saccharomyces cerevisiae* is a powerful model organism to study autophagy. Yeast is simple to cultivate, and its genome is fully sequenced and can easily be modified. In yeast, bulk autophagy is inhibited in nutrient-rich conditions. This facilitates studying early steps in bulk autophagy upon its induction by starvation or rapamycin treatment and to investigate selective autophagy separately from bulk autophagy. Numerous methods to study autophagy are well established, many of which do not require specialized equipment. They allow researchers to address questions with more than one approach and to confirm obtained results using independent readouts. As the core proteins and mechanisms are conserved from yeast to mammals, knowledge gained from autophagy research in yeast can often be transferred to mammalian cells. Thus, mechanisms elucidated in yeast help to identify potential drug targets in human diseases linked to autophagy.
